# Soil macro-fauna respond to environmental variations along a coastal-inland gradient

**DOI:** 10.7717/peerj.9532

**Published:** 2020-07-14

**Authors:** Xiaoxue Zheng, Yan Tao, Zhongqiang Wang, Chen Ma, Hong He, Xiuqin Yin

**Affiliations:** 1 Key Laboratory of Geographical Processes and Ecological Security in Changbai Mountains, Ministry of Education, School of Geographical Sciences, Northeast Normal University, Changchun, Jilin Province, China; 2 Jilin Provincial Key Laboratory of Animal Resource Conservation and Utilization, Changchun, Jilin Province, China; 3 School of Public Administration and Law, Northeast Agricultural University, Harbin, Heilongjiang Province, China; 4 School of Natural Resources, University of Missouri, Columbia, MO, USA

**Keywords:** Soil macro-fauna, Community characteristics, Coastal ecosystems, Coastal-inland gradient, Bohai Bay

## Abstract

Varied environmental conditions in coastal-inland zones tend to influence soil faunal communities. However, few studies have focused on the responses of soil fauna to environmental variations along the coastal-inland gradient. In order to better understand the aforementioned responses, a total of 80 soil macro-faunal samples were collected at the five different distances from the coastline of China’s Bohai Bay. The results revealed that the compositions, structural characteristics and diversity of the soil macro-fauna varied among the different habitats. With the increases in the distance from the sea, the individual density, richness and diversity levels of the soil macro-fauna all first increased and then decreased. The individual density, richness and diversity values were all at their maximum at 30 km from the sea. The Edge effect promoted unique and rare soil macro-faunal taxa. Formicidae, Curculionidae and Aphodiidae were found to be the edge taxa. Agelenidae, Liocranidae and Nematocera were considered to be indicator taxa of severe sea effects. Paradoxosomatidae was an indicator taxon of slight effects. Overall, the environmental variations along the coastal-inland gradient were found to have the potential to affect the soil macro-faunal communities, and the different taxa of the soil macro-fauna responded to those variations in different ways. This study further revealed the processes and mechanisms of the sea influencing the soil macro-faunal communities, which had been caused by the coastal-inland gradient. The results of this study also provided a theoretical basis for developing future biodiversity guidelines for coastal ecosystems.

## Introduction

Coastal ecosystems, the joints of entire terrestrial ecosystems and oceanic ecosystems, are characterized by active natural processes and vulnerable ecosystems (Holligan & Reiners, 1992). The belowground system in the coast is clearly different from inland, and is significantly affected by tides, sea winds, salinity and ocean current (Langodan et al., 2020; Boer, Rydberg & Saide, 2000). Sea winds or storm surges can cause seawater floods on beaches, subsequently increasing soil moisture in the coast, and this gradually decreases along the coastal-inland gradient (Dodet et al., 2019). The tides can raise or reduce levels of coastal groundwater, thereby altering the soil texture and structure of the coast (Idier et al., 2019). Ba & Zhao (1997) have reported that, due to the fact that the effect of tides on the coast gradually declines along the coastal-inland gradient, sandy soil is typically found near the seashore, whereas loam or clay can be collected from the further offshore. Soil salinization is a common phenomenon in the belowground system of coasts (Wang et al., 2007). Seawater intrusion and exchanges between seawater and groundwater can accumulate redundant salt in the soil (Khanom, 2016; Werner et al., 2013). Dítě et al. (2019) have observed clear variations among vegetation communities along the coastal-inland gradient. Species-poor vegetation was found on the mudflat zone characterized by high salinity, while the highest richness of vegetation occurred in the upper tidal zone with the lowest soil salinity. Based on these observations, the coastal-inland gradient is crucial for affecting soil nutrient and properties, and thus it is necessary to provide some insight into elucidating these effects on belowground system.

Due to the weak tolerance and poor ability to migrate, the taxonomic compositions and distribution patterns of soil fauna will sensitively respond to environmental changes (Meloni et al., 2020; Yin et al., 2019; Tao et al., 2019). It is known that the environment in the coast is always changing (Liang et al., 2020), and thus soil fauna provide valuable indicators of environmental changes in the coast. At present, most studies regarding responses of soil fauna to environmental variations have mainly focused on the diversity and spatiotemporal patterns of soil fauna. Balakrishnan, Srinivasan & Mohanraj (2014) have reported that the diversity of some soil fauna varied in the different coastal habitats of Tamil Nadu, India (Balakrishnan, Srinivasan & Mohanraj, 2014). Ghosh & Mandal (2018) have found that the free-living nematodes can respond to environmental changes in the tropical coasts (Ghosh & Mandal, 2018). The spatiotemporal patterns of earthworm community were shown to indicate different habitats in the coastal grass of West Africa (Rossi & Lavelle, 2015; Jaikishun et al., 2015). Soil fauna were found to exhibit the natural and anthropogenic effects on the west coast of North American, and the most influential natural factors on the abundance and composition of soil fauna were found to be vegetation conditions (David et al., 2016). However, few studies concentrating on the responses of soil faunal communities to the environmental variations caused by the coastal-inland gradient.

As the only semi-closed sea in China, the Bohai Bay has an advantageous geographical location and a typical coastal ecosystem. Therefore, the Bohai Bay was considered to be an ideal area for conducting in-depth study of the coastal-inland gradient. In order to provide information regarding the responses of soil macro-faunal communities to environmental variations along the coastal-inland gradient, soil macro-faunal samples were collected at the different distances from the coastline of the Bohai Bay, that is, 1 km, 15 km, 30 km, 45 km and 60 km. We hypothesized that (H1) the composition and diversity of soil macro-faunal communities would differ along the coastal-inland gradient, due to the changes in environmental factors; and (H2) the soil macro-faunal taxa would differ in response to the effects of coastal-inland gradient.

## Materials and Methods

### Study area

**Figure 1:**
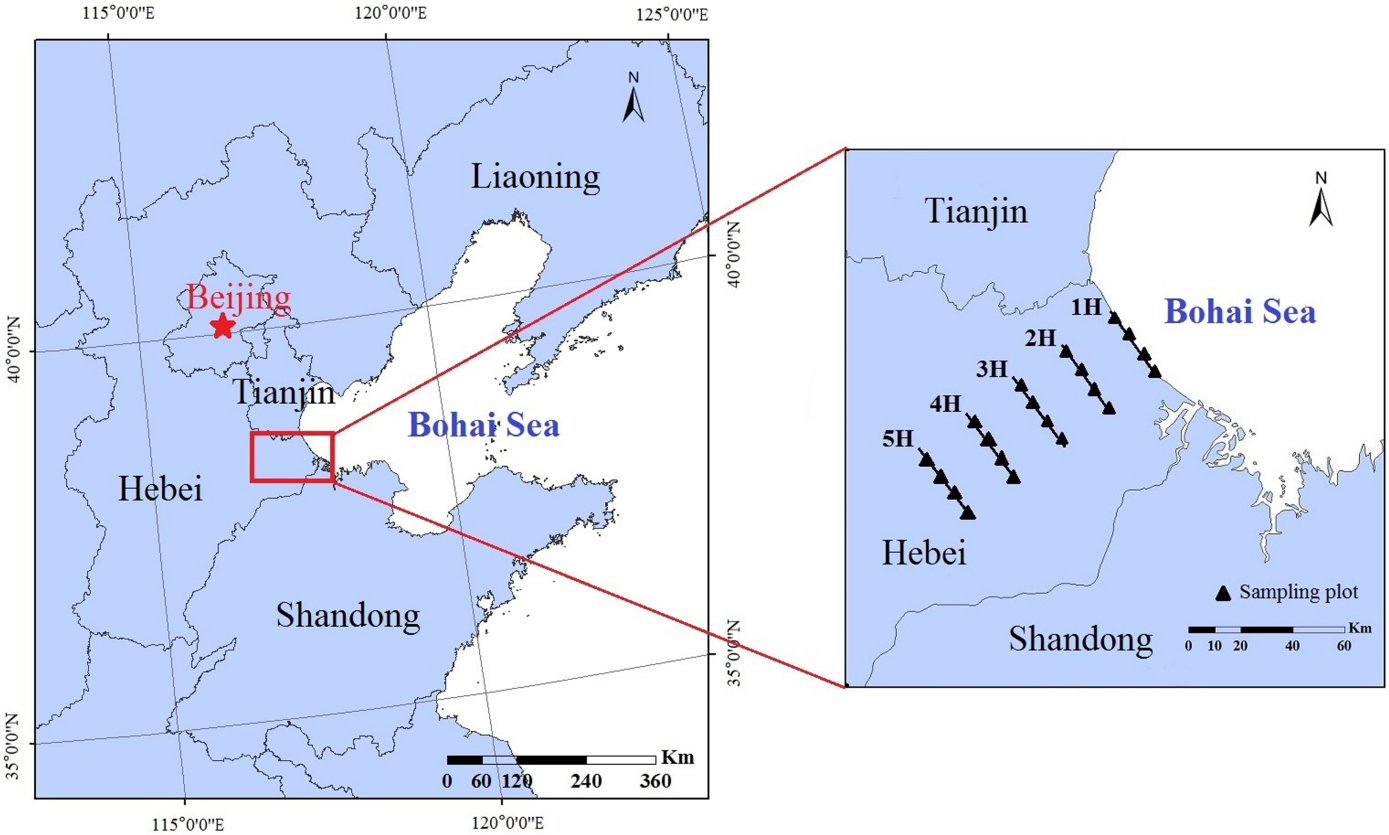
Locations of the experimental sites and sampling lines in China’s Bohai Bay area. 1H, 2H, 3H, 4H and 5H represent the sampling lines; and the black solid triangles represent the sampling plots.

The experiments in this study were carried out in the coastal areas of the Bohai Bay, which is located in Huanghua, Hebei Province, China (38°09′-38°39′N;117°05′-117°40′E) (Fig. 1). The region situated in the western section of the Bohai Bay has a warm temperate marine monsoon climate, which gradually converts to a warm temperate continental monsoon climate as it extends inland (Hu et al., 2013; Zou et al., 2011). The annual average temperature is 12.2 °C. The annual precipitation is approximately 500 mm and is mainly concentrated during the summer months. The soil type of the study is mainly saline-alkali soil, with a soil salinity reaching nearly 0.5% (Gao et al., 2016). The dominant vegetation species were determined to be *Phragmites australis*, *Puccinellia tenuiflora* and *Artemisia scoparia*.

### Sampling design

In order to correspond to the period of vegetation growth, soil-faunal samples were collected in July (summer) of 2019. From the coastline to the inland areas, five sampling lines were established according to the definition of a coastal zone, that is, a low-land extending 100 km to the mainland from the coastline (Board of Millennium Ecosystem Assessment, 2001) and the pre-experimental results. The distance ranges were 1 km (Sampling line 1H); 15 km (Sampling line 2H); 30 km (Sampling line 3H); 45 km (Sampling line 4H); and 60 km (Sampling line 5H). The geographical information and vegetation characteristics of each sampling line are shown in Table 1. In this study, within each sampling line, four 20 × 20 m sampling plots were established, and an interval of at least 1 km was set between each pair of sampling plots in order to ensure their independence (Fig. 1). In each sampling plot, four subplots measuring 50 cm (length) × 50 cm (width) × 20 cm (depth) were randomly established. All of the soil macro-fauna samples were collected using a hand-sorting method, and then preserved in a 75% alcohol solution. A total of 80 soil faunal samples were collected as follows: 5 sampling lines × 4 sampling plots × 4 subplots. Then, all of the collected soil macro-faunal specimens were counted using an OLYMPUS SZX16 stereoscopic microscope, and identified at the family (or suborder) level (Yin, 2000a, 2000b). In accordance with the classification system of soil faunal taxa, the taxa in which individuals accounted for more than 10% of the total individuals of soil macro-fauna were defined as dominant taxa. Subsequently, the taxa with individuals which accounted for 1% to 10% of the total individuals were defined as common taxa, and those with individuals which accounted for less than 1% of the total individuals were considered to be rare taxa (Xu, 2018).

**Table 1:** Geographical information and vegetation characteristics of the five sampling lines.

	Sampling lines				
	1H	2H	3H	4H	5H
Distance from Bohai (km)	1.00	15.00	30.00	45.00	60.00
Elevation (m)	2.00	2.00	3.00	3.00	7.00
Dominant vegetation species	*Phragmites australis*,	*Phragmites australis*	*Phragmites australis*, *Puccinellia tenuiflora*, *Artemisia scoparia*	*Phragmites australis*, *Artemisia scoparia*, *Sonchus wightianus*	*Phragmites australis*, *Imperata cylindrica*

In each subplot, soil samples measuring 10 cm (length) × 10 cm (width) × 20 cm (depth) were collected and transported to this study’s laboratory facilities for physical and chemical soil analyses. After removing the leaves, roots and gravels from the soil samples, all of the soil samples were air-dried and stored under indoor temperature conditions. The aboveground plant biomass (PB) was harvested from 100 cm × 100 cm squares localized in each sampling plot (Minden et al., 2016). The plant coverage (PC) was directly estimated by the visual estimation method (Sutherland, 1999). All plant material was oven-dried at 80 °C for at least 24 h before being weighed (Leadley, Niklaus & Stocker, 1999). The physical and chemical properties of the soil were determined using conventional methods. The soil moisture levels were measured using an oven drying method (Schmugge, Jackson & Mckim, 1980). The soil pH was determined using 1:2.5 soil/water method. The soil organic matter was digested using K_2_Cr_2_O_7_–H_2_SO_4_ and examined using FeSO_4_ titration (Sims & Haby, 1971). In addition, total N in the soil was digested by H_2_SO_4_ and K_2_SO_4_–CuSO_4_–Se (Tel & Jansen, 2008). The available P of the soil was analyzed using a molybdenum spectrophotometer method after extraction with NaHCO_3_ (Iatrou et al., 2014). The available K of the soil was determined using a flame photometer after the soil samples were extracted with 1 mol/L NH_4_OAc (soil: solution 1:10) (Lu et al., 2017).The soil cation exchange capacity (CEC) was determined by 1 mol/L NH_4_OAc, which was then expressed as cmol/kg air-dried soil (Savvides et al., 2010). The concentration of Na^+^ in the soil was determined using a flame photometer apparatus (Richards, 1954). Table 2 details the soil properties in the five sampling lines.

**Table 2:** Soil properties and vegetation characteristics in the five sampling lines (mean ± SE).

	Sampling lines				
	1H	2H	3H	4H	5H
SM (%)	27 ± 2.97a	21 ± 2.34b	20 ± 1.71b	19 ± 0.27b	15 ± 0.96c
SOM (g/kg)	13.29 ± 0.29b	14.06 ± 0.15b	17.07 ± 0.62a	11.99 ± 0.45c	10.95 ± 0.12c
TN (mg/kg)	610.11 ± 7.47a	625.51 ± 3.08a	645.37 ± 9.16a	552.12 ± 2.29b	517.89 ± 1.50b
AP (mg/kg)	13.63 ± 0.71b	19.53 ± 0.77a	23.78 ± 0.46a	15.07 ± 0.58b	7.31 ± 0.49c
AK (mg/kg)	272.03 ± 3.10a	291.40 ± 1.88a	297.51 ± 1.92a	233.04 ± 3.08b	211.64 ± 2.47b
CEC (cmol/kg)	14.57 ± 1.86a	14.22 ± 1.03a	14.16 ± 1.36a	13.20 ± 1.32b	13.19 ± 0.31b
pH	8.26 ± 0.11a	8.24 ± 0.08a	8.22 ± 0.13a	8.25 ± 0.10a	8.23 ± 0.06a
Na^+^ (mg/L)	132.40 ± 3.05a	129.27 ± 2.50b	128.68 ± 0.45b	119.98 ± 1.10c	119.93 ± 2.71c
PB (kg/m^2^)	84.92 ± 2.95c	109.15 ± 2.23b	171.84 ± 2.72a	147.32 ± 1.79b	145.73 ± 7.62b
PC (%)	60 ± 3.00c	65 ± 2.50c	86 ± 4.25a	81 ± 4.00a	76 ± 4.20b

**Notes:**

SM, soil moisture; SOM, soil organic matter; TN, soil total N; AP, soil available P; AK, soil available K; CEC, soil cation exchange capacity; PB, plant biomass; PC, plant coverage.

The least significant difference (LSD) was used to compare the means; values with different letters (a, b and c) indicate means with significant differences at *p* < 0.05.

### Statistical analysis

In the present study, in order to analyze the effects of distance from the coastline on the composition and diversity of the soil macro-faunal communities, the soil macro-faunal taxa of each sampling line were manually determined. A Venn diagram was created using a “draw-quintuple-Venn” function, which was available in a Venn Diagram R package (Chen, 2018). In addition, running SPSS 22 (SPSS Inc., Chicago, IL, USA), generalized linear models (GLMs) were used to determine the environmental variations along the coastal-inland gradient on individual diversity levels, taxonomic compositions and general diversity levels of the soil macro-faunal communities.

Then, the diversity of the soil macro-fauna was quantitatively analyzed using the Shannon-Wiener index (H′) (Weaver & Shannon, 1949) as follows:

$$H^\prime = - \mathop \sum \limits_{i = 1}^s {P_i}\ln {P_i}$$

where *S* is the number of taxa; and *P*_*i*_ is the ratio of individuals to the total individuals in taxon *i*.

A cluster analysis with the “hclust” function, which was available in the stats R package, was used to analyze the similarities among the different soil macro-faunal communities of each sampling line (R Development Core Team, 2019). A heat map of the hierarchical clustering was created for the purpose of evaluating the distribution patterns of the different soil macro-faunal taxa, which were available in the pheatmap R package and vegan R package (Raivo, 2019; Oksanen et al., 2016).

In addition, a redundancy analysis (RDA) process was applied in order to analyze the correlations between the dominant and common taxa of soil macro-fauna and the variations in the environmental factors caused by the coastal-inland gradient. The data sets of redundancy analysis included the dominant and common soil macro-faunal taxa, and the following environmental factors: Soil moisture; soil organic matter; soil total N; soil available P; soil available K; soil CEC; Na^+^ plant biomass; and plant coverage. Also, the redundancy analysis was performed in Canoco and CanoDraw for Windows (Lepš & Šmilauer, 2014; Lai, 2013).

## Results

### Taxonomic compositions of the soil macro-faunal communities

In this study, a total of 10,656 soil macro-fauna individuals, belonging to 38 taxa (families/suborders), were collected from the five sampling lines. The dominant taxa of the soil macro-fauna were found to be Formicidae (55.48%) and Trachelipidae (15.84%). In addition, Staphylinidae (6.12%), Carabidae (2.29%), Gnaphosidae (2.29%), Aristocera (2.27%) and Scaphidiidae (2.14%) were considered to be common taxa, which together accounted for 15.11% of the total individuals. The other 31 taxa were found to be infrequent, and together accounted for 13.57% of the total individuals of the soil macro-fauna.

**Figure 2:**
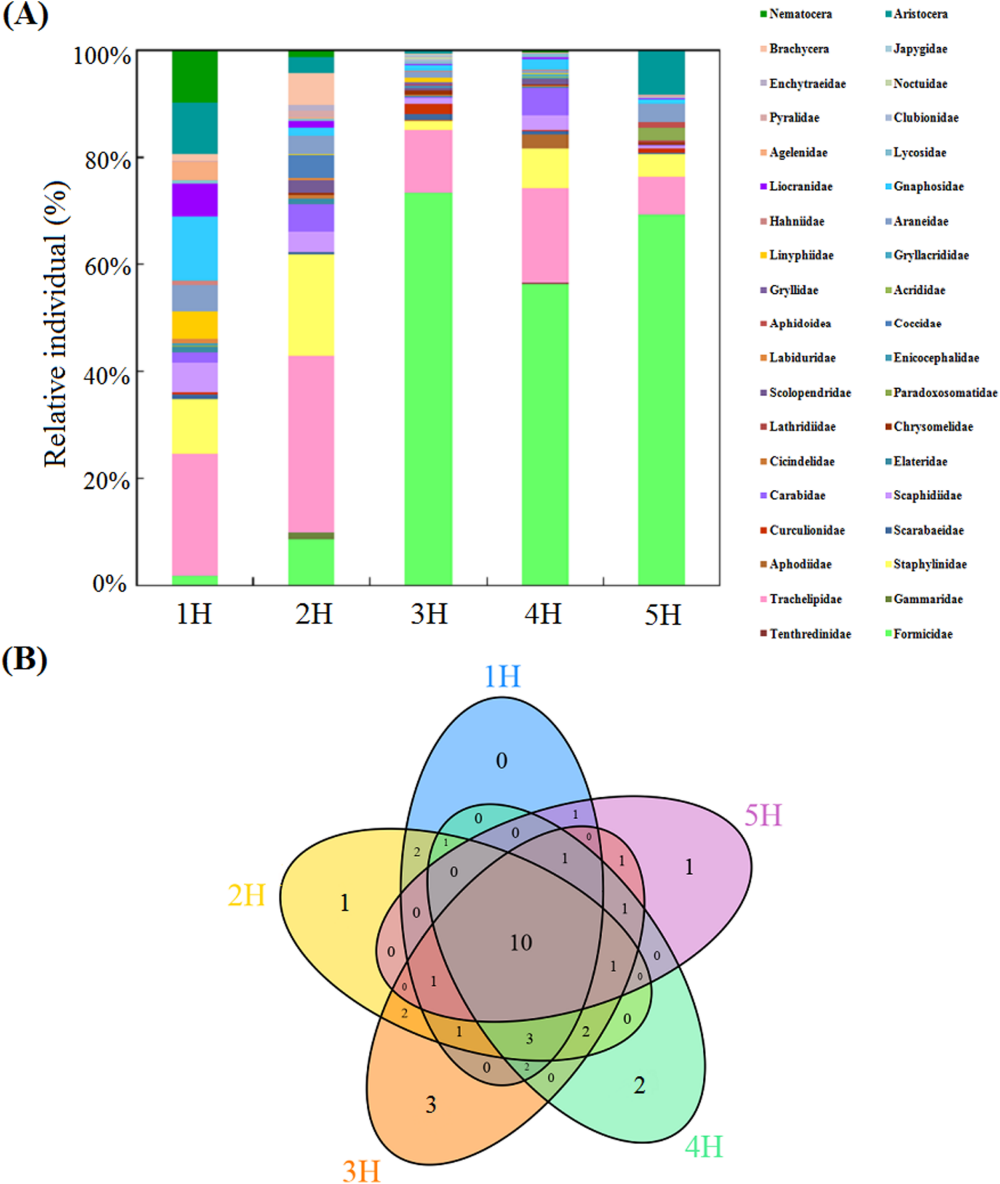
Compositions of the soil macro-faunal communities in the five sampling lines. (A) Column diagram of the relative individual of the soil macro-faunal communities; (B) Venn diagram of the number of shared and unique soil macro-faunal taxa, the shared and unique numbers within the circles indicate the number of either shared taxa or unique taxa in the overlapping regions.

The taxonomic compositions of the soil macro-faunal communities in the five sampling lines are displayed in Fig. 2A and Table S1. In the sampling line of 1H, the dominant taxa of the soil macro-fauna were found to be Trachelipidae (22.50%), Gnaphosidae (12.08%) and Staphylinidae (10.42%). In the 2H sampling line, Trachelipidae (32.65%) and Staphylinidae (19.18%) were the dominant taxa of the soil macro-fauna. In the sampling lines of 3H and 4H, Formicidae and Trachelipidae were frequently collected, which together accounted for 85.16% and 74.26% of each total individuals of soil macro-fauna, respectively. However, only the Formicidae (69.42%) was found to be the dominant taxon of the soil macro-fauna in the 5H sampling line.

This study’s Venn diagram is detailed in Fig. 2B, in which the shared and unique taxa of soil macro-fauna can be seen. The five sampling lines had ten shared taxa (Formicidae, Trachelipidae, Staphylinidae, Scarabaeidae, Scaphidiidae, Carabidae, Araneidae, Gnaphosidae, Liocranidae and Pyralidae), which contributed to between 67.08% and 92.70% of the full set of taxa in each sampling line. With the increases in the distance from the sea, the unique taxa of soil macro-fauna in each sampling line first increased and then decreased. In the 1H sampling line, the unique taxa of the soil macro-fauna were not observed. Only one unique taxon (Gammaridae) was found in the 2H sampling line. In the sampling line of 3H, the unique taxa were observed to be Gryllidae, Japygidae and Noctuidae. In the 4H sampling line, the unique taxa were Tenthredinidae and Gryllacrididae. Lathridiidae was only observed in the 5H sampling line.

### Distribution characteristics of the soil macro-faunal communities

In this study, the distribution characteristics of soil macro-faunal communities were found to be different at the five different sampling distances. The information regarding the soil macro-fauna individuals is summarized in Table 3 and Table S1. The individual density of soil macro-fauna in the 3H sampling line was the largest (1,044 individuals/m^2^), and was significantly greater than those of the other sampling lines (*p* < 0.05). The smallest individual density was found in the 1H sampling line (240 individuals/m^2^), and there were no significant differences observed between the 1H and 2H sampling lines.

**Table 3:** Individual density (individuals/m^2^), richness and Shannon–Wiener Index of the soil macro-faunal communities in the five sampling lines.

	Sampling lines					
	1H		2H	3H	4H	5H
Individual density	240 ± 2c		245 ± 2c	1044 ± 5a	808 ± 4b	327 ± 3c
Richness	22		24	30	25	17
Shannon-Wiener Index	1.29 ± 0.06c		1.59 ± 0.07b	2.53 ± 0.16a	2.36 ± 0.13a	1.22 ± 0.06c

**Note:**

The lower case letters (a, b and c) indicate the significant differences in each sampling line within the same individual density and Shannon–Wiener Index at the *p* < 0.05 level.

The sequencings of the richness and Shannon–Wiener Index were as follows: 3H > 4H > 2H > 1H > 5H (Table 3). Compared with the sequencing of the individual densities, the individual density of the soil macro-fauna in the 5H sampling line was larger than those of the 1H and 2H sampling lines. However, the richness and diversity levels were lower than those of the 1H and 2H sampling lines.

**Figure 3:**
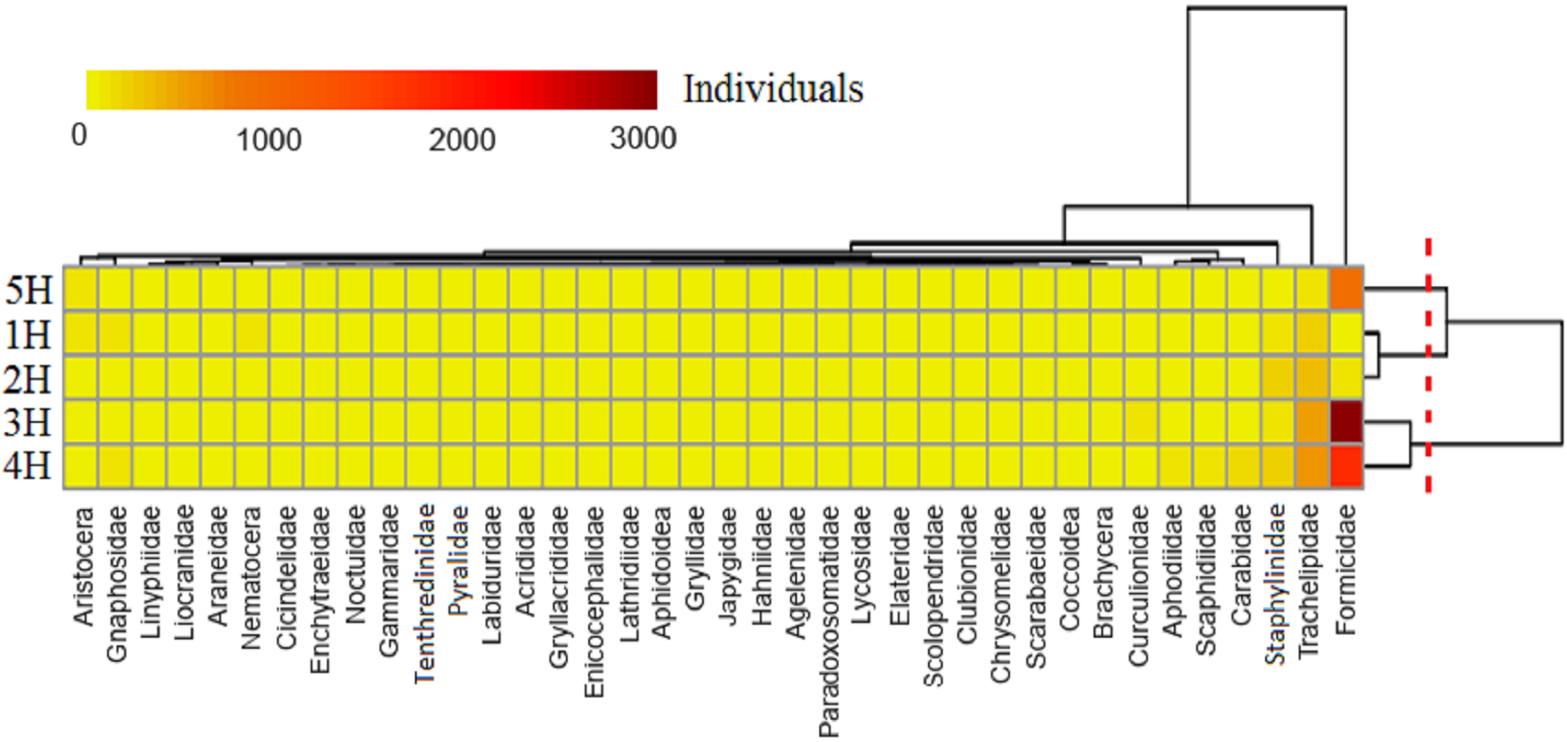
Heat map of the soil macro-fauna individuals in the five sampling lines. Dendrogram of the sampling lines based on similarity along the right axis; dendrogram of the soil macro-faunal taxa based on similarity along the upper axis. The colors represent the individuals of the soil macro-fauna.

A heat map displayed that the five sampling lines could be divided into three clusters (Fig. 3): the 5H sampling line cluster; 3H and 4H sampling lines cluster; and a cluster of the 1H and 2H sampling lines. It indicated that the environmental conditions in the 1H and 2H sampling lines were very similar to one another. This was also evident for the 3H and 4H sampling lines, and all of the sampling lines differed from the 5H. The taxa of the soil macro-fauna could be divided into four clusters: Formicidae, Trachelipidae and Staphylinidae composed three clusters, respectively, and the other taxa made up the final cluster. A greater number of Formicidae was found in sampling lines of 3H, 4H and 5H. The majority of Trachelipidae was distributed in the 3H and 4H. In addition, Staphylinidae was mainly found in the 2H. In contrast, the distributions of the other soil macro-faunal taxa were observed to be relatively rare in the coastal habitats of the Bohai Bay.

### Correlations between the coastal environmental factors and the soil macro-faunal taxa

**Figure 4:**
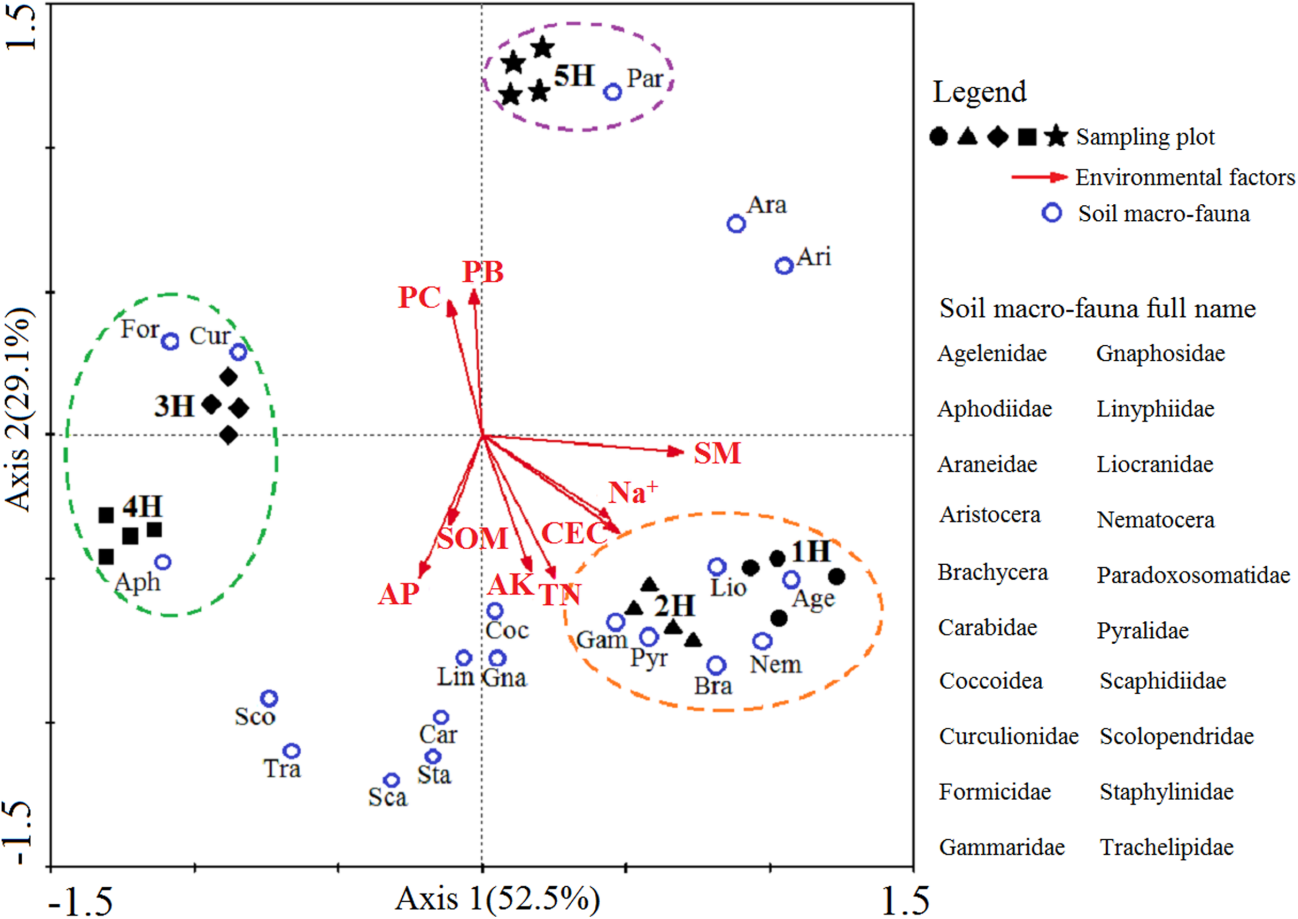
Two-dimensional diagram of redundancy analysis (RDA). The black solid symbols represent the sampling plots corresponding to each sampling line; The red arrowed lines represent the environmental factors, and their abbreviation meanings are listed in Table 2; the small blue circles indicate the dominant and common soil macro-faunal taxa, and their full names are listed in the right column of RDA diagram; The percentages appearing next to the axis numbers are the total variations of the data explained by that specific axis.

A two-dimensional diagram of the redundancy analysis (RDA) was completed to determine the correlation between the dominant and common soil macro-faunal taxa and the environmental variations caused by the coastal-inland gradient (Fig. 4). Axis 1 and Axis 2 explain 52.5% and 29.1% the integrated information of the sequencing objects (sampling lines), responsive variables (soil macro-faunal taxa) and explanatory variables (environmental factors), respectively. The diagram demonstrated the responses of soil macro-faunal taxa to the environmental variations. It was found that the Agelenidae, Liocranidae, Nematocera, Brachycera, Pyralidae and Gammaridae were positive within the 1H and 2H sampling lines, and had responded positively to soil moisture, soil CEC, Na^+^, soil total N and soil available K, but negatively to the plant biomass and plant coverage. Formicidae, Curculionidae and Aphodiidae had positively responded to the 3H and 4H sampling lines, but correlated negatively to the soil moisture, soil CEC and Na^+^. Paradoxosomatidae had responded positively to the 5H sampling line, and was found to be positively correlated with the plant biomass and plant coverage. However, it had a negative relationship with the soil organic matter and soil available P.

## Discussion

### Habitat-derived shifts in community composition of soil macro-fauna

In this study, we found that the composition and diversity of soil macro-faunal communities differed within each sampling distance, which was caused by the changes in environmental factors along the coastal-inland gradient, and this was in line with our hypothesis (H1) as predicted. The individual density (1,044 individuals/m^2^), richness (30 taxa) and diversity index (2.53) in the 3H sampling line were significantly greater than those of the other sampling lines. The Edge effect causes the changes in population or diversity that occur at the boundary of two habitats (Kroodsma, 1984; Laurance, Didham & Power, 2001). In general, density and diversity of animals is greater in the ecotone between the different habitats (Smith et al., 1997; Grover & Wilbur, 2002). The 3H sampling line was set up at intermediate distance from the sea, and thus it could be considered as the ecotone between the inland and coast. Consequently, the individual density, richness and diversity of the soil macro-faunal communities were all at the maximum.

By contrast, the 5H sampling line was located at the farthest distance from the sea, at almost 60 km away. The effects of sea in the 5H sampling line were the lowest, due to the fact that the intensity levels of sea winds, sea waves and tides were significantly weakened. However, the effects of inland were greater with the increases in distance from the sea, thus causing the characteristics of the environmental conditions in the 5H sampling line to be similar to those of an inland environment. The richness and diversity in the 5H sampling line were observed to be the lowest. This was determined to be due to the fact that the lower effects of the sea resulted in the habitat of the 5H sampling line becoming more homogeneous (Zeng et al., 2017). Therefore, it was concluded that the homogeneity of the environmental conditions in the 5H sampling line had decreased the richness and diversity of the soil macro-faunal communities to some extent.

The individual densities of the soil macro-fauna in the 1H and 2H sampling lines were smaller than those in the other sampling lines. Due to the nearness to the sea, 1H and 2H had experienced the maximum effects associated with the sea, such as sea winds, wave and tidal disturbances (Boer, Rydberg & Saide, 2000). It was clear that the closer to the sea, the higher the intensity and frequency of sea winds, which resulted in growth restrictions in natural vegetation. These restrictions subsequently negatively affected the food sources of the soil macro-faunal subsistence (Zheng, Pan & Li, 2013). In addition, under the impacts of sea waves and tides, groundwater level remained fluctuating in the coastal aquifer. Sea waves and tides increased the exchanging frequency of the moisture and salt at the interfaces between seawater and groundwater, which resulted in active groundwater cycles in the inshore zones (Morrissey et al., 2014), subsequently increasing the contents of soil salinity, and reducing the coastal vegetation biomass and coverage. As a result, soil macro-faunal individual densities were declined in the 1H and 2H sampling lines.

### Specific responses of different taxa to the variations in environmental factors

It was observed in this study that the different soil macro-faunal taxa in the different sampling lines responded to the variations in environmental factors caused by the coastal-inland gradient in various ways, which confirmed this study’s second hypothesis (H2). As shown in Fig. 4, due to discrepancies in the nutrition methods, life histories and adaptability mechanisms of soil macro-faunal taxa, the sea had impacted the soil macro-faunal taxa to different extents.

This study’s results demonstrated that the sampling plots in the five sampling lines had various environment factor characteristics, as detailed in Table 2 and Fig. 4. Among the soil physicochemical properties, the soil moisture and soil CEC levels were found to have positive correlations with the 1H and 2H sampling lines. These findings indicated that the soil environments in the 1H and 2H sampling lines were much wetter. However, it was found that with the increases in the distance from the sea, the content levels of the soil moisture, along with the soil salinity, were significantly reduced. The soil CEC refers to the ability of the cation adsorbed by soil colloid, which can be affected by soil textures and organic matter content levels (Bortoluzzi et al., 2006). The values of soil CEC basically represent the amount of nutrients held by the soil. In other words, the soil CEC values represent the soil fertility levels. In the present study, the content levels of the soil nutrients were observed to be higher in the 1H and 2H sampling lines. In contrast, the soil moisture and soil CEC values were found to have negative correlations with the 3H and 4H sampling lines. However, the soil organic matter, plant biomass and plant coverage all had positive correlations with the aforementioned two sampling lines. It was also observed that natural plants, such as *Phragmites australis* and *Puccinellia tenuiflora*, extensively covered soil surface in those areas, which provided abundant food sources and comfortable habitats for the growth and reproduction of the soil macro-fauna. Finally, it was observed in the 5H sampling line that the soil moisture and soil CEC values were relatively moderate, and the environmental condition in the 5H sampling line tended to have the characteristics of a neutral habitat.

In the 1H and 2H sampling lines, the soil macro-faunal taxa were mainly types of taxa which had successfully adapted to a wet soil environment. The dominant and common taxa were Agelenidae, Liocranidae, Nematocera, Gammaridae, Pyralidae and Brachycera, which are known to adapt well to the inshore habitats. However, this study also found that only the Agelenidae, Liocranidae and Nematocera displayed regular gradient changes in which their individuals decreased along the coastal-inland gradient (Table S1). These changing trends of the aforementioned three soil macro-fauna individuals may be explained by their diet characteristics and life-history traits. For example, the Agelenidae and Liocranidae are both predatory taxa, and the Nematocera is categorized as a type of omnivore (Michalko & Košulič, 2020; Steinwandtera et al., 2018). In the 1H and 2H sampling lines, the soil macro-faunal taxa were required to adapt to the inshore regions with lower plant biomass. The predatory and omnivorous characteristics of the previously mentioned soil macro-faunal taxa allowed them to meet their food source requirements and maintain their normal growth and reproduction rates. Therefore, the presence of those three soil macro-faunal taxa were considered to be positive indicators of severe effects from the sea.

The positive soil macro-faunal taxa within the 3H and 4H sampling lines were Formicidae, Curculionidae and Aphodiidae. These taxa types were found to have adapted well to the habitat conditions of lower soil moisture, soil CEC and Na^+^. Formicidae is considered to be a type of omnivore, with such nutritional sources as leaves, seeds, small insects and nectar. The majority of the Curculionidae feed exclusively on plants, including seeds, stems, flower heads and roots (Barratt, Cock & Oberprieler, 2018). The Aphodiidae is mainly detritivore, feeding on plant residue or dung produced by various animals (Finch et al., 2020; Buse et al., 2018). Generally speaking, the aforementioned three soil macro-faunal taxa could potentially survive on plants or plant residues. In the 3H and 4H sampling lines, the abundant plant biomass and extensive plant coverage provided sufficient food sources for the growth and reproduction of the three taxa. In the present study, the individuals of the Formicidae, Curculionidae and Aphodiidae were significantly higher in the 3H and 4H sampling lines. Therefore, the three taxa were regarded as the edge taxa. Additionally, Na^+^ was essential for most of the soil fauna decomposers (Kaspari, Welti & Beurs, 2020; Krumm et al., 2005), but may not for soil fauna predators, thus the changes of Na^+^ content in soil and litter would change the soil fauna communities along the coastal-inland gradient.

Located at the farthest distance from the sea, the 5H sampling line’s environmental conditions, along with the compositions of the soil macro-faunal communities, were found to be more similar to those of the inland area. Paradoxosomatidae was found to be obviously positive within the 5H habitat. It was found that the individuals of Paradoxosomatidae increased significantly along the coastal-inland gradient, with the maximum number observed in the 5H sampling line (Table S1). The Paradoxosomatidae is a variety of terrestrial arthropod, which mainly feed on plant residue, such as decaying plant matter and rotten wood (Rodrigues et al., 2017). It has been found that by living on soil surfaces or under soil clods, the Paradoxosomatidae plays a vital role in litter decomposition within local habitats. Therefore, in the present study, the Paradoxosomatidae was considered to be an indicator taxon of slight effects caused by the sea.

## Conclusions

In summary, the compositions, structural characteristics and diversity of the soil macro-fauna varied along the coastal-inland gradient. The Edge effect tended to occur at approximately 30 km from the sea, where the individual density, richness levels and diversity rates of the soil fauna were all at the maximum. Extending inland from the distance of 30 km from the sea, the effects of sea were found to decrease, the environmental conditions and soil faunal communities were observed to be more similar to those of the inland areas. From the distance of 30 km to the coastline, the effects of sea were noticeably increased, and the environmental conditions and soil faunal communities were observed to be similar to those of the inshore habitats. Soil faunal taxa responded to the variations of environmental factors caused by the coastal-inland gradient. The different types of soil faunal taxa indicated the effects intensities of the sea in various ways. The findings obtained in this study have implications for deepening the current understanding of the ecological significance of soil fauna and could potentially provide assistance in developing biodiversity guidelines for coastal ecosystems in the future.

## Supplemental Information

10.7717/peerj.9532/supp-1 Supplemental Information 1 Individuals of soil macro-fauna in the habitats of the five sampling lines.

## Additional Information and Declarations

### Data Availability

The following information was supplied regarding data availability: The raw measurements are available as a Supplemental File.
